# Job control and working life expectancy in Sweden

**DOI:** 10.5271/sjweh.4250

**Published:** 2025-11-01

**Authors:** Melody Almroth, Alicia Nevriana, Daniel Falkstedt, Alex Burdorf, Katarina Kjellberg, Tomas Hemmingsson, Kuan-Yu Pan, Jacob Pedersen

**Affiliations:** 1 Institution of Environmental Medicine, Karolinska Institutet, Stockholm, Sweden.; 2 Department of Public Health, Erasmus Medical Center, Rotterdam, Netherlands.; 3 Centre for Occupational and Environmental Medicine, Region Stockholm, Stockholm, Sweden.; 4 Department of Public Health Sciences, Stockholm University, Stockholm, Sweden.; 5 National Research Center for Work Environment, Lersø Parkallé, Copenhagen, Denmark.

**Keywords:** labor market participation, psychosocial working condition, working years lost

## Abstract

**Objectives:**

This study aimed to investigate the impact of low job control on labor market participation expressed through working life expectancy (WLE) and working years lost (WYL) among men and women in Sweden.

**Methods:**

A random sample of 100 000 individuals was drawn from the Swedish Work, Illness, and labor market Participation (SWIP) cohort of the registered Swedish population in 2005 born 1945 to 1975. The multi-state estimated labor market affiliation method was used to estimate WLE and WYL due to unemployment, sickness absence, other, disability pension, early old-age pension, and death over a 15-year period (2006–2020). Job control was assessed through a job exposure matrix.

**Results:**

Men and women in high-control jobs had a longer WLE at each age. At age 30, the WLE for men in high-control jobs was 26.3 years while for men in low-control jobs this was 2.5 years shorter. For women, WLE at 30 was 25.8 years for high-control jobs but nearly five years shorter for low-control jobs. For both men and women, these differences were mostly due to disability pension and unemployment. Those in lower control jobs could expect to lose more working years according to nearly all other states besides active employment.

**Conclusions:**

Higher job control is linked to longer WLE, while low job control is an important determinant of WYL in the Swedish workforce. Addressing low job control could extend working lives and reduce inequities in labor market outcomes.

Due to population aging, attempts are being made to extend working life in all European countries, including Sweden (1). However, many are not able to work until the current normative retirement age (65 years old) (2). This is especially the case for those in blue-collar jobs, which tend to be characterized by poorer physical, psychosocial, and organizational working conditions (3–5).

It is therefore important to investigate labor market patterns among those with different working conditions to understand where improvements can be made to extend working life. Calculating working life expectancy (WLE) – the estimated average number of years a group of people is expected to remain in the workforce – and working years lost (WYL), which captures the potential years of lost workforce participation due to reasons like unemployment, sickness absence, disability, and early retirement, makes it possible to understand the overall impact of labor market policies (6). Stemming from the well-known concept of life expectancy, WLE uses observed information to make predictions based on probabilities (7). This approach allows for accounting for labor market transitions across working life by estimating the duration of labor force exit rather than just the likelihood of an event.

Low job control has emerged as one important organizational work factor in predicting negative singular labor market outcomes such as sickness absence (8) and disability pension (9–12). Low job control is characterized by low influence over decisions at work and limited possibilities to develop new skills in relation to work (13). A few previous Swedish studies have looked at job control in relation to multiple later labor market positions. These studies found that low job control was related to sickness absence, disability pension and unemployment cross-sectionally (14) as well as longitudinally (15), with the latter showing additional but unclear results for early pension.

One previous study found high job control to be related to a longer healthy life expectancy (16), which is a related but different concept than WLE. Other studies have considered WLE, but according to socioeconomic status rather than specific working conditions. These studies have found that those with lower levels of education and in manual occupational classes have a shorter WLE (2, 17–21). A recent study estimated WLE according to level of job strain (the combination of low job control and high job demands) and found that those without job strain had a longer WLE (22). However, this study relied on panel data collected every two years and only estimated time in and out of work without considering specific reasons for being outside of the labor market. An understanding of how job control relates to WLE and WYL due to specific reasons such as sickness absence and unemployment could be valuable, as job control may help explain differences in these states and represents a factor that can potentially be modified through intervention.

To the best of our knowledge, no previous study has investigated job control in relation to WLE or WYL. The aim of the present study was to investigate the impact of low job control on labor market participation expressed through WLE and WYL among men and women in Sweden.

## Methods

### Study population and data

This study is based on the Swedish Work Illness and labor market Participation (SWIP) cohort, which includes the registered Swedish population aged 16–64 years in 2005 (around 5.4 million). The present is restricted to individuals born 1945–1975 who were alive at the start of the year 2006, had not received disability pension prior to the age of 30, and had a registered occupation in 2006 (N=3 111 823). A random sample of 100 000 individuals was used for the analyses. Linked information from the total population register (23), the longitudinal integrated database for health insurance and labor market studies (LISA) register (24), the micro data for social insurance (MIDAS) register, the national patient register (25), the pension authority register, and the employment agency register are used. Parental socioeconomic background information and occupational level working conditions are also linked to the index persons.

The Regional Ethics Review Board in Stockholm approved the study (reference number 2017/1224-31, 2018/1675-32, and 2022/02725-02).

### Measures

*Job control.* This was measured using a job exposure matrix (JEM) based on around 90 000 responses to the Swedish Work Environment surveys that were administered every second year from 1997 to 2013 in 355 occupations. This JEM is based on four questions on decision authority and three on skill discretion. The questions on decision authority measure the respondent’s ability to determine which tasks to do, the pace of their work, when to take breaks, and the structure of their work (26). The questions on skill discretion measure opportunities for learning and development, problem solving, and repetitive work (27). Recent publications have shown the predictive validity of this JEM for, eg, depression, disability pension, and mortality (12, 26, 27).

The mean of the seven items over the entire period is calculated for each occupation for men and women separately and linked to the index person based on their registered occupation in 2006 which is recorded as the SSYK-96, [the Swedish version of the International Standard Classification of Occupations (ISCO-88)]. Each occupation was then assigned to a sex-specific tertile using the JEM, based on the total population distribution.

Supplementary material (www.sjweh.fi/article/4250) table S1 shows the ten most common occupations according to sex and job control level.

*Labor market affiliation.* The Swedish welfare system compensates time outside of gainful employment through several different systems. The social insurance system consists of disability pension and sickness absence compensation, where disability pension is for people of working age who have been assessed as not being able to work full-time again due to illness or disability (24), while sickness absence is considered temporary and paid by the employer for the first 14 days and covered by the social insurance agency thereafter (24). In situations where the person does not have an employer (ie, if they are unemployed or self-employed), then the sickness absence is covered by the social insurance system from the beginning of the period. The unemployment insurance fund (*arbetslöshetkassa* or *A-kassa*) provides benefits for individuals who are seeking work and ready to take a suitable job (24). Public pension funds are accumulated throughout working life based on income and could be withdrawn at age 61 at the earliest for the individuals included in the present study (28). Based on these different systems, individuals’ labor market affiliation was tracked over a period of 15 years (from the start of 2006 until the end of 2020 or until their 65^th^ birthday) and defined as seven mutually exclusive states.

Three states were treated as absorbing states, meaning that once a transition was made into one of these states, no further transitions are observed. These states include *death, early old-age pension, and disability pension* with relevant dates obtained from the total population register, the pension authority register, and the MIDAS register respectively.

Four states were treated as recurrent states, meaning that individuals could transition between these states. Information on periods of *unemployment* was obtained through the employment agency register. *Sickness absence* periods of ≥14 days were defined through the MIDAS register of the social insurance agency. We estimated *time in work* based on the condition that the person was not in any of the other states and had an income of at least one price base amount (PBA) during the corresponding calendar year (29). The state “*other*” represents the time that individuals were not in any of the other defined states and also not registered as earning an income of one PBA during the corresponding calendar year. This could include periods of parental leave, education, migration from Sweden, or relying on other income such as from other assets or a spouse. The PBA is calculated by Statistics Sweden every year to determine insurance and retirement levels. It tends to be around US$5000–6000 during an entire year.

If an individual was in more than one state on a given day (for example, if they were on part-time sickness absence), their state was prioritized based on the following hierarchy: 1=death, 2=pension, 3=disability pension, 4=sickness absence, 5=unemployment, 6=work, and 7=other.

### Covariates

Several covariates were chosen that could differ according to job control level and be related to labor market position. Legal sex was measured from the total population register. Country of birth was attained from the LISA register and categorized to indicate whether the individual was born in Sweden or not.

Socioeconomic position during childhood was measured by linking the index person to their parents’ census information from 1960, 1970, or 1980, when the index person was 5–15 years old. Occupational information was taken primarily from the father, but when this information was missing, the mother’s occupation was used. The parents’ occupations were then classified as non-manual employee at a higher level, non-manual employee at an intermediate level, assistant non-manual employee, skilled manual worker, non-skilled manual worker, farmer, and those with no parental occupation reported (30).

Previous morbidity was measured by identifying the presence of any diagnosis included in the Charlson co-morbidity index (CCI) in the national inpatient registers dating back as early as the 1960s. These diagnoses include myocardial infarction, congestive heart failure, peripheral vascular disease, cerebrovascular disease, pulmonary diseases, rheumatic disease, dementia, hemiplegia, diabetes, chronic kidney disease, liver disease, peptic ulcer disease, cancer, and HIV/AIDS (31). This was categorized as a binary variable to indicate whether the individual had a history of any of the included diagnoses.

The highest attained education in 2006 was taken from the LISA register and was categorized based on total years of education (≤9, 10–11, 12, 13–14, and ≥15 years of education). These categories range from compulsory school only to ≥3 years of university education.

Marital status was obtained from the total population register in 2006 and categorized as unmarried, married, divorced, or widowed. The number of children <20 years living in the household was obtained from the LISA register in 2006 and categorized as 0, 1, 2, 3, and ≥4.

Employment sector was measured in the LISA register in 2006 and categorized as either public or private sector. Physical workload was measured using a JEM based on eight survey items regarding heavy lifting, uncomfortable working postures, repetitive work, and physically demanding work. A gender-specific index mean value for these eight items was calculated to assess the occupational overall physical exposure. This value was linked to individuals based on their registered occupation in 2006 and categorized into tertiles based on the population distribution.

### Statistical analysis

The distribution of covariates was first compared according to sex in both the source and sample population.

**Figure 1:**
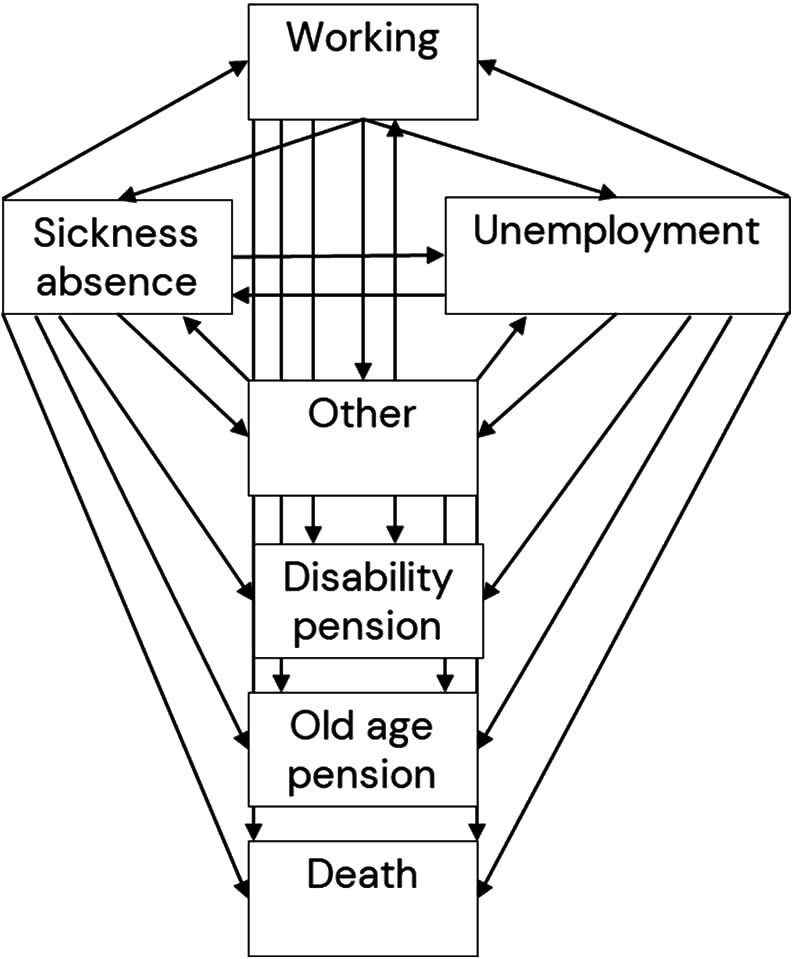
The seven possible states and 24 possible transitions.

We estimated WLE and the state-specific WYL using a long format arrangement of the date-based individual register-based follow-ups (32) and the ELMA method developed by Pedersen et al (6, 33). The ELMA method relies on multiple Cox regression analysis on the transition probabilities between the possible labor market-related states of the multi-state model (see figure 1). The ELMA method incorporates left and right censoring, time truncation, recurrent events, competing events management, covariates, and weights while fulfilling a Markov assumption (34). We used age as the time axis and performed integrations of the transition probability curves to estimate the WLE and WYL for four different starting ages with the reference group of employees with medium job control. Depending on the starting age, we then used estimates from Cox proportional hazards regression to gain the transitions probability curves of the references group (35, 36).

We estimated the area under the curves and used bootstrapping for simulating the variance. We then used simple variance analysis to find the variable-specific contribution of having low and high job control compared to the reference (35). All covariates except job control, sex, and age were included as inverse probability weights. Due to limitations in computational power, analyses were performed on a random sample of 100 000 individuals. Analyses were done in SAS V.9.4 and graphs were created using R V4.4.1.

### Supplementary and sensitivity analyses

Data retrieved from the pension authority and the employment agency are not commonly used in Swedish register-based studies but include more detailed information on date and duration of pension and unemployment. To assess the quality of the data, we compared these registers to the known information included in the commonly used LISA register.

To test the robustness of using a random sample, we repeated the analyses on three different random samples of 100 000. We also decreased the sample size gradually to see if results remained stable.

To better understand the role of the covariates used in the analysis, we explored the distribution of each covariate according to level of job control for men and women separately. We also repeated analyses without adjusting for any covariates.

Because job control and physical workload are highly inversely correlated (Spearman correlation=0.78), it is difficult to completely disentangle their independent effects. We therefore repeated the analyses excluding those with high physical workload.

## Results

The distribution of covariates during 2006 was similar for men and women except for women being slightly higher educated and more likely to have children living at home and men being more likely to be unmarried and to work in the private sector (table 1). We found no substantial deviations in the distribution of covariates between the total source population and the random sample of 100 000 persons on which the main analysis is based.

**Table 1:** Distribution of baseline (2006) characteristics of the source (SWIP) and sample population according to sex. [SEP=socioeconomic position; CCI=Charlson co-morbidity index]

		Source				Sample		
		N=3 111 823				N=100 000		
		Men		Women		Men		Women
		N (%)		N (%)		%		%
Total		1 547 293 (49.7)		1 564 530 (50.3)		50.8		49.2
Age (years)								
	31–40	526 915 (34.1)		512 340 (32.8)		34.3		33.0
	41–50	502 350 (32.5)		510 384 (32.6)		32.3		32.6
	51–61	518 028 (33.5)		541 806 (34.6)		33.5		34.5
Birth country								
	Outside Sweden	184 040 (11.9)		203 203 (13.0)		11.8		12.8
Childhood SEP								
	Unskilled manual	409 855 (26.5)		411 366 (26.3)		26.6		26.5
	Skilled manual	321 596 (20.8)		324 199 (20.7)		21.1		20.7
	Lower non-manual	159 257 (10.3)		158 131 (10.1)		10.3		10.2
	Intermediate non-manual	272 712 (17.6)		267 394 (17.1)		17.6		16.8
	Professional non-manual	92 698 (6.0)		90 464 (5.8)		5.9		5.9
	Farmer	83 520 (5.4)		87 808 (5.6)		5.4		5.5
	Not classified	207 637 (13.4)		225 168 (14.4)		13.2		14.3
CCI								
	Previous diagnosis	88 776 (5.7)		90 374 (5.8)		5.7		5.7
Education								
	<9 years	254 910 (15.9)		165 575 (10.6)		16.3		10.7
	10–11 years	528 313 (34.2)		502 268 (32.1)		34.1		31.9
	12 years	242 266 (15.7)		250 342 (16.0)		15.5		16.1
	13–14 years	226 446 (14.7)		259 183 (16.6)		14.6		16.7
	>15	300 412 (19.5)		385 173 (24.7)		19.5		24.6
Marital status								
	Married	785 714 (50.8)		842 814 (53.9)		50.5		53.3
	Unmarried	571 484 (36.9)		455 930 (29.1)		37.1		29.6
	Divorced	181 876 (11.8)		241 120 (15.4)		11.9		15.4
	Widowed	8 172 (0.5)		24 650 (1.6)		0.5		1.7
Children								
	0	812 042 (52.5)		728 103 (46.5)		52.5		46.8
	1	249 464 (16.1)		300 640 (19.2)		16.1		19.2
	2	350 337 (22.6)		388 987 (24.9)		22.9		24.6
	3	109 897 (7.1)		120 557 (7.7)		6.9		7.7
	≥4	15 553 (1.7)		26 243 (1.7)		1.6		1.7
Sector								
	Private	1 108 695 (76.4)		603 669 (42.6)		76.3		42.5
Job control								
	Low	514 114 (33.0)		565 977 (36.1)		33.3		36.2
	Medium	510 831 (33.0)		479 815 (30.7)		33.0		30.5
	High	522 348 (33.8)		519 736 (33.2)		33.8		33.3
Physical workload								
	Low	493 415 (31.9)		528 828 (33.8)		31.8		33.7
	Medium	538 116 (34.8)		527 950 (33.7)		34.8		33.9
	High	515 762 (33.3)		507 752 (32.5)		33.4		32.4

**Figure 2:**
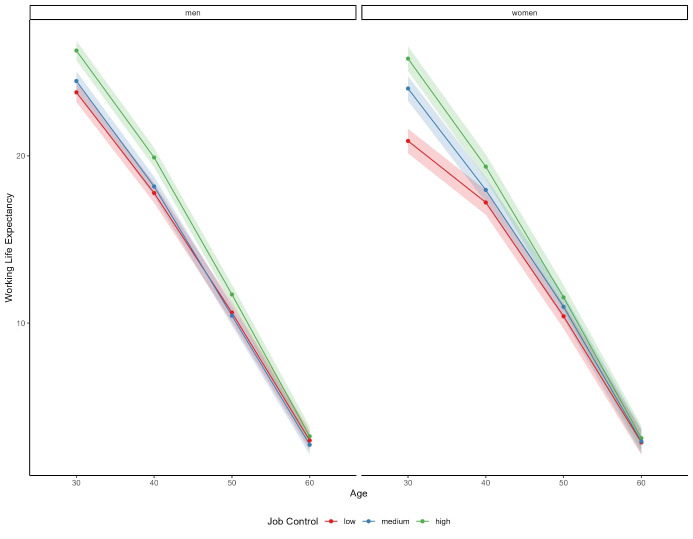
Working life expectancy (and 95% confidence) intervals according to sex, age, and level of job control.

Generally, men had a slightly higher WLE compared to women at each age (figure 2). Those with high job control had a higher WLE across age bands and among both men and women. Men with high job control at age 30 could expect 2.5 additional working years compared to men with low job control and for women this difference was nearly 5 years [26.3 years, 95% confidence interval (CI) 25.7–26.9 for men in high-control jobs compared to 23.8 years, 95% CI 23.2–24.4 for men in low-control jobs and 25.8 years, 95% CI 25.1–26.6 for women in high-control jobs compared to 20.9 years, 95% CI 20.2–21.6 for women in low-control jobs] (table 2). At age 40, men and women in high compared to low-control jobs could expect to work 2.12 and 2.14 years longer, respectively. At age 50, this was 1.08 and 1.13 and, at age 60, 0.25 and 0.26 years. For both men and women, these differences were mostly due to disability pension and unemployment.

**Table 2:** Expected average years spent in different states up to 65 years of age and 95% confidence intervals by sex, age, and level of job control adjusted for birth country, childhood socioeconomic position, Charlson co-morbidity index, education level, marital status, number of children, employment sector, and occupational physical workload. [CI=confidence interval; WLE=working life expectancy]

Age		Job control	Work		Sickness absence		Unemployed		Other		Disability pension		Early pension		Death
			WLE (95% CI)		WLE (95% CI)		WLE (95% CI)		WLE (95% CI)		WLE (95% CI)		WLE (95% CI)		WLE (95% CI)
Men															
	30	Low	23.80 (23.21–24.38)		1.29 (1.11–1.47)		3.23 (2.99–3.47)		3.35 (3.05–3.65)		1.35 (1.17–1.53)		0.57 (0.53–0.60)		0.66 (0.61–0.71)
		Medium	24.47 (23.88–25.06)		1.22 (1.04–1.40)		2.82 (2.58–3.05)		3.27 (2.97–3.57)		1.37 (1.18–1.55)		0.92 (0.88–0.95)		0.49 (0.44–0.54)
		High	26.29 (25.71–26.88)		1.03 (0.85–1.21)		1.84 (1.61–2.08)		3.26 (2.96–3.56)		0.24 (0.06–0.42)		0.65 (0.61–0.69)		0.45 (0.40–0.50)
	40	Low	17.78 (17.19–18.36)		0.65 (0.47–0.83)		2.12 (1.88–2.36)		2.33 (2.03–2.63)		0.59 (0.41–0.77)		0.59 (0.55–0.63)		0.49 (0.44–0.54)
		Medium	18.17 (17.58–18.75)		0.77 (0.59–0.95)		1.50 (1.26–1.73)		2.13 (1.83–2.43)		0.91 (0.73–1.09)		0.91 (0.87–0.95)		0.38 (0.33–0.43)
		High	19.90 (19.31–20.49)		0.58 (0.39–0.76)		0.86 (0.62–1.10)		2.42 (2.12–2.72)		0.17 (-0.01–0.35)		0.63 (0.6–0.67)		0.34 (0.29–0.39)
	50	Low	10.63 (10.04–11.21)		0.51 (0.33–0.69)		1.04 (0.80–1.27)		1.50 (1.20–1.80)		0.36 (0.18–0.54)		0.61 (0.57–0.64)		0.25 (0.20–0.31)
		Medium	10.44 (9.86–11.03)		0.58 (0.40–0.76)		0.70 (0.46–0.93)		1.36 (1.06–1.66)		0.58 (0.40–0.76)		0.96 (0.92–0.99)		0.21 (0.16–0.26)
		High	11.71 (11.13–12.3)		0.41 (0.23–0.59)		0.49 (0.25–0.72)		1.45 (1.15–1.76)		0.11 (-0.07–0.29)		0.65 (0.61–0.69)		0.14 (0.09–0.20)
	60	Low	2.98 (2.39–3.57)		0.18 (0.00–0.36)		0.34 (0.11–0.58)		0.73 (0.43–1.03)		0.09 (-0.09–0.27)		0.66 (0.62–0.7)		0.04 (-0.02–0.09)
		Medium	2.72 (2.14–3.31)		0.17 (-0.01–0.35)		0.28 (0.05–0.52)		0.48 (0.18–0.78)		0.06 (-0.12–0.24)		1.01 (0.97–1.04)		0.03 (-0.03–0.08)
		High	3.23 (2.65–3.82)		0.11 (-0.07–0.29)		0.18 (-0.06–0.42)		0.72 (0.42–1.02)		0.03 (-0.15–0.21)		0.67 (0.63–0.71)		0.03 (-0.03–0.08)
Women															
	30	Low	20.89 (20.15–21.63)		1.91 (1.70–2.11)		3.87 (3.59–4.16)		3.90 (3.56–4.24)		3.09 (2.67–3.52)		0.59 (0.53–0.64)		0.23 (0.19–0.28)
		Medium	24.02 (23.28–24.76)		2.01 (1.81–2.21)		2.07 (1.79–2.36)		3.32 (2.98–3.66)		3.76 (3.33–4.19)		0.84 (0.78–0.9)		0.16 (0.12–0.21)
		High	25.81 (25.07–26.55)		1.91 (1.71–2.12)		1.99 (1.71–2.27)		3.61 (3.27–3.95)		1.69 (1.26–2.11)		0.62 (0.56–0.68)		0.25 (0.20–0.29)
	40	Low	17.21 (16.47–17.95)		1.50 (1.30–1.70)		1.84 (1.56–2.13)		2.45 (2.11–2.79)		1.30 (0.87–1.73)		0.65 (0.59–0.71)		0.21 (0.16–0.25)
		Medium	17.96 (17.22–18.70)		1.38 (1.17–1.58)		0.97 (0.69–1.25)		1.65 (1.31–1.99)		1.79 (1.36–2.22)		0.92 (0.86–0.98)		0.20 (0.15–0.24)
		High	19.35 (18.61–20.09)		1.34 (1.13–1.54)		1.01 (0.72–1.29)		2.06 (1.72–2.40)		0.68 (0.26–1.11)		0.61 (0.55–0.67)		0.13 (0.08–0.17)
	50	Low	10.41 (9.67–11.15)		0.83 (0.63–1.04)		0.97 (0.69–1.26)		1.40 (1.06–1.74)		0.58 (0.15–1.01)		0.70 (0.64–0.76)		0.09 (0.04–0.13)
		Medium	10.97 (10.23–11.71)		0.92 (0.72–1.13)		0.52 (0.23–0.80)		1.03 (0.69–1.37)		0.59 (0.17–1.02)		0.94 (0.88–1)		0.07 (0.03–0.12)
		High	11.54 (10.8–12.28)		0.74 (0.54–0.94)		0.50 (0.22–0.79)		1.32 (0.98–1.66)		0.23 (-0.20–0.65)		0.57 (0.51–0.63)		0.09 (0.04–0.13)
	60	Low	2.86 (2.12–3.60)		0.21 (0.01–0.41)		0.24 (-0.04–0.53)		0.77 (0.43–1.11)		0.06 (-0.37–0.49)		0.75 (0.69–0.81)		0.01 (-0.03–0.06)
		Medium	2.93 (2.19–3.67)		0.21 (0.00–0.41)		0.20 (-0.09–0.48)		0.56 (0.22–0.90)		0.09 (-0.34–0.52)		0.99 (0.93–1.05)		0.01 (-0.03–0.06)
		High	3.12 (2.38–3.86)		0.16 (-0.05–0.36)		0.24 (-0.04–0.53)		0.81 (0.47–1.15)		0.05 (-0.38–0.47)		0.62 (0.56–0.68)		0.00 (-0.04–0.05)

**Figure 3:**
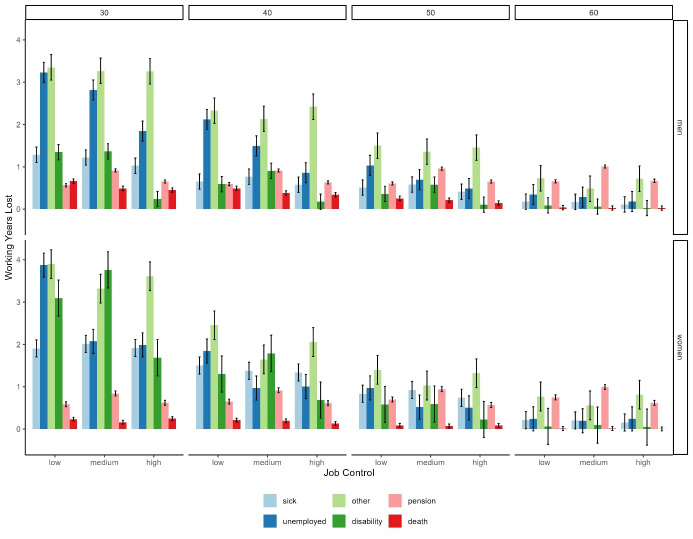
Expected working years lost due to sickness absence, unemployment, other, disability pension, early old age pension, and death (with 95% confidence intervals) according to sex, age, and level of job control.

Women were expected to lose more working years due to sickness absence and disability pension compared to men, while men could expect to lose slightly more working years due to death. Men and women could expect similar WYL due to unemployment, early pension, and “other” (figure 3 and table 2).

For men, the greatest expected losses in working years due to sickness absence, unemployment, and death were found among those in low-control jobs, while the greatest losses in working years due to disability pension and early pension were found among men in medium-control jobs. For women, the greatest WYL due to unemployment were found among those with low job control while the greatest WYL due to sickness absence, disability pension, and early pension were found among those in medium-control jobs. For both men and women, those in medium-control jobs could expect to lose the least amount of WYL due to “other”.

### Results of sensitivity analyses

When investigating the quality of the unemployment and pension registers, we found that the days of unemployment reported in the employment agency register were higher than those in the LISA register around 10% of the time in the first years of follow-up, but that this discrepancy decreased over time (supplementary table S2). The year of pension was nearly perfectly correlated in the pension authority and LISA registers (supplementary table S3).

We also found that when we performed the same analyses on three different random samples of 100 000 individuals, results were statistically identical (not shown). When we gradually decreased the sample size, the results became less stable.

Job control correlated with several important covariates. For example, those with low job control were more likely to be born outside of Sweden, had worse health and lower socioeconomic position and higher physical workload (supplementary table S4). Substantial differences were found when comparing the unadjusted estimates to estimates where covariates were included, indicating the importance of adjusting in the main analyses (supplementary table S5).

We found consistent patterns when excluding those with high physical workload from analyses, though overall WLE was more similar between medium and low job control levels (supplementary figures S1 and S2).

## Discussion

This study found that men and women with high job control had a higher WLE than those in lower-control jobs, demonstrating an exposure–response pattern at each age. Those in lower control jobs could expect more WYL due to nearly all states outside of active labor. For the state “other”, those in medium-control jobs could expect to lose the least working years.

Our results are in line with several previous studies that have shown low job control to be related to an increased risk in a variety of negative labor market outcomes including sickness absence and disability pension (8–12, 14, 15). However, these studies did not calculate WLE or WYL, making them difficult to compare directly. Previous studies from Finland (17), The Netherlands (19), and a multi-national study including Sweden (18) show educational inequalities in WLE and WYL. However, these studies tend to show larger age- and sex-specific educational differences than the job control differences shown in the present study. Because job control is tied to employment, those without occupations were excluded from the present study, likely narrowing exposure group differences. A Finnish study found a one-year WLE gap at age 50 between manual and non-manual employees, similar to our own findings (20). Similar differences in WLE have also been found according to physical exposure level (20, 35).

A recent Swedish study found that those with job strain (low job control and high job demands) had a WLE at age 50 that was around 0.5 years shorter than those without job strain (22), which is slightly lower than in the present study. In our own previous studies, we have found that high job demands tend to predict more positive health and labor market outcomes (12, 26) and, thus, may mask some of the consequences of low job control when categorized together as “job strain”.

Having higher job control has several advantages for extending working life, which have previously been discussed within the job strain model (13). The possibility to make decisions about how work is done may be key in managing stress and achieving a better work–life balance. Having more opportunities to learn and develop may be inherent in more stimulating and meaningful jobs which promote a longer working life. Jobs with high levels of control may also have more favorable working conditions in general, which leads to a more secure labor market position and better health. Those who end up in occupations with higher levels of job control may be healthier and more advantaged in other ways, though the present results accounted for several such factors including socioeconomic background, education, and physical workload. A recent meta-review presented compelling evidence that organizational-level interventions addressing job control could lead to improved health but also pointed to the need for further research to better understand the implementation of such interventions (37).

The differences between men and women in whether low or medium job control led to the highest expected WYL due to sickness absence and disability pension may reflect gendered patterns in illness severity and access to benefits. Higher WYL due to early pension in medium-control jobs could result from workers with reduced workability who do not qualify for disability pension. Higher WYL in the “other” category among high- and low-control workers likely reflects different reasons for labor market absence. Those with high job control may take longer parental leave, pursue education, work abroad, or rely on other assets, while those with low job control may experience weaker labor market attachment and rely more on a partner’s income.

### Strengths and limitations

The strengths of this study include being able to draw from a nationally representative sample of over three million individuals with 15 years of detailed labor market information. Information provided by the pension authority and employment agency extends what has previously been possible using annual information only. This is also the largest study using the ELMA method, which provides more precise estimates compared to previous methods of measuring WLE (34). This allowed the possibility of adjusting for covariates, which has rarely been possible previously. Quantifying WLE and WYL provides more intuitive results compared to relative risk measures.

This study also has some limitations. SWIP is a closed cohort, so estimates at age 30 are based only on those aged 30 at the start of the observation. Due to the computationally intensive calculations, analyses were performed on a random sample of 100 000 rather than the entire population. However, the sample used in the study was representative of the whole population, and repeated analyses on different random samples showed statistically identical results, indicating that this was a sufficient sample size.

JEM provide objective occupational exposure measures but can lead to non-differential misclassification due to the lack of individual-level variation. It is also difficult to disentangle occupational exposures as they tend to be highly correlated. Though we accounted for physical workload, we cannot completely disentangle co-existing exposures and occupational conditions. Because we did not have complete information on job control for the entire follow-up period, job control and the other covariates were estimated in 2006 and applied to the entire observation period. Though potential changes in exposure levels during the follow-up period were not accounted for, we have found these to be consistent over time in the same source population (27).

Using annual income to categorize work and non-work likely overestimated work periods, as we could not fully account for periods of parental leave and studying. Furthermore, we did not account for individuals being in more than one state at a time (eg, part-time sickness absence combined with work). Both early pension and disability pension were treated as absorbing states, though returning to work is possible. For disability pension, this concerned around 2% of cases, but for pension this is unclear since individuals can combine their withdrawal of pension with gainful employment. A previous study using the SWIP cohort found that around 10–15% of the eligible population combined early pension with income from work (38). However, the extent and timing of work is not possible to decipher because only annual income is reported, so this was not accounted for in the present study.

We included the Charlson co-morbidity index to adjust for health, though it may involve some over adjustment, as poor health may be on the causal pathway between job control and labor market position, as individuals are expected to have already been in their occupations for some time at baseline. Finally, WLE is a predictive measure and relies on the assumption that underlying economic conditions are stable.

### Concluding remarks

Men and women in high-control jobs can expect to have longer working lives, while those with lower job control can expect to lose more years of working life, particularly to disability pension, unemployment and sickness absence. These differences in expected labor market position according to level of job control represent inequities in society, where those with a longer WLE can expect better life-long income and pension. Improving opportunities for influence and development at work could be one way to extend working life and reduce inequities. Organizational interventions aimed at improving job control have shown promising evidence, and further improving these strategies and their implementation would be an important direction for future research.

## Supplementary material

Supplementary material
