# Supplementary material for: Job control and working life expectancy in Sweden
Source: Scand J Work Environ Health. 2025 Oct 30;51(6):516–25. doi: 10.5271/sjweh.4250 (PMC12592985; doi:10.5271/sjweh.4250)
Supplement: Supplementary material [file SJWEH-51-516-S001.pdf]

Job control and working life expectancy in Sweden[1]

by Melody Almroth, PhD,[2] Alicia Nevriana, PhD, Daniel Falkstedt, PhD, Alex Burdorf, PhD, Katarina Kjellberg, PhD, Tomas Hemmingsson, PhD, Kuan-Yu Pan, PhD, Jacob Pedersen, PhD

1. Supplementary material
2. Correspondence to: Melody Almroth, Institution of Environmental Medicine, Karolinska Institutet. Solnavägen 4, 11365 Stockholm, Sweden. [E-mail: melody.almroth@ki.se]

Table S1 the ten most common occupations according to sex and job control level during 2006

Sex	Job control	Code	Occupational title	N
**Men**	**Low**		**Total**	514,114
		8323	Heavy truck and lorry drivers	41,952
		8211	Machine tool operators	32,793
		4131	Store clerks and storekeepers	31,863
		8290	Other machine operators and assemblers	17,465
		5133	Home based personal care and related workers	17,258
		8322	Bus and tram drivers	17,036
		9190	Other sales and services elementary occupations	15,625
		7212	Welders and flame cutters	13,740
		8321	Car, taxi and van drivers	13,515
		9320	Manufacturing laborers	13,343
	**Medium**		**Total**	510,831
		7123	Carpenters and joiners	33,152
		7137	Building caretakers	30,930
		5222	Shop salespersons non-food stores	21,233
		7231	Motor vehicle mechanics and fitters	20,074
		7233	Agricultural- or industrial-machinery mechanics and fitters	18,818
		2330	Primary education teaching professionals	18,652
		1314	Managers of small enterprises in wholesale and retail trade, hotels and restaurants, transport and communications	18,354
		4190	Other office clerks	15,649
		2221	Medical doctors	15,637
		7136	Building and related electricians	15,265
	**High**		**Total**	522,348
		3415	Technical and commercial sales representatives	51,053
		2131	Computer systems designers, analysts and programmers	40,255
		3119	Physical and engineering science technicians not elsewhere classified	27,363
		3121	Computer assistants	21,218
		2470	Public service administrative professionals	18,337
		1210	Directors and chief executives	18,116
		3115	Mechanical engineering technicians	16,762
		3112	Civil engineering technicians	15,289
		2310	College, university and higher education teaching professionals	14,903
		3114	Electronics and telecommunications engineering technicians	14,504
**Women**	**Low**		**Total**	564,977
		5132	Assistant nurses and hospital care and related workers	131,055
		5133	Home-based personal care and related workers	106,054
		9122	Helpers and cleaners in offices, hotels and other establishments	49,590
		5222	Show salespersons, non-food stores	32,692
		9130	Helpers in restaurants	31,155
		5221	Shop salespersons, food stores	26,057
		5122	Cooks	16,055
		4222	Receptionists	11,698
		5135	Dental nurses	10,685
		9190	Other sales and services elementary occupations	9,628
	**Medium**		**Total**	479,815
		5131	Child-care workers	73,489
		4190	Other office clerks	67,383
		3310	Pre-primary education teaching associate professionals	66,622
		2330	Primary education teaching professionals	59,408

	3239	Nursing associate professionals not elsewhere classified	37,796
	4112	Office secretaries	29,805
	3418	Banking associate professionals	14,646
	2340	Special education teaching professionals	11,147
	2323	Teaching professionals, artistic and practical subjects	11,146
	3443	Bookkeepers	10,890
**High**		**Total**	519,738
	4120	Numerical clerks	43,685
	5134	Attendants, psychiatric care	39,946
	3431	Administrative secretaries and related associate professionals	27,049
	2470	Public service administrative professionals	26,611
	2492	Social work professionals	19,344
	3415	Technical and commercial sales representatives	18,060
	3433	Bookkeepers	14,732
	2411	Accountants	14,412
	2221	Medical doctors	12,653
	2310	College, university and higher education teaching professionals	11,942

Table S2 comparison of days of unemployment between unemployment agency register and LISA register across years in the SWIP population

	2006 %	2007 %	2008 %	2009 %	2010 %	2011 %	2012 %	2013 %	2014 %	2015 %	2016 %	2017 %	2018 %	2019 %
**LISA=UA**	87.8	89.9	91.2	90.4	89.8	91.1	91.8	92.1	93.0	93.8	94.4	95.1	95.7	96.3
**UA>LISA**	11.7	9.7	8.6	9.2	9.9	8.6	7.9	7.7	6.8	6.0	5.4	4.7	4.1	3.3
**LISA>UA**	0.5	0.4	0.2	0.4	0.3	0.3	0.3	0.2	0.2	0.2	0.2	0.2	0.2	0.4
**<30 days***	1.3	1.2	1.2	1.1	0.9	0.8	0.7	0.7	0.6	0.5	0.5	0.4	0.4	0.4
**31-60 days***	1.4	1.2	1.0	1.1	1.0	0.8	0.8	0.7	0.6	0.6	0.5	0.4	0.4	0.4
**>60 days***	9.5	7.8	6.7	7.4	8.4	7.2	6.8	6.6	5.8	5.2	4.6	4.0	3.5	2.9

UA=Unemployment Agency.

*Difference in days between the two registers

Table S3 comparison of year of pension between pension authority register and LISA register across years in the entire SWIP population

	**LISA year**														
**PA year**	**None**	**2006 %**	**2007 %**	**2008 %**	**2009 %**	**2010 %**	**2011 %**	**2012 %**	**2013 %**	**2014 %**	**2015 %**	**2016 %**	**2017 %**	**2018 %**	**2019 %**
**None**	99.58	0.17	0.13	0.05	0.01	0	0	0	0	0	0.01	0.01	0.03	0.02	0.49
**2006**	0.02	98.44	0.19	0.01	0.01	0.01	0.01	0.01	0.01	0	0	0	0	0	0
**2007**	0.02	1.32	98.68	0.08	0.01	0.01	0.01	0.01	0.01	0.01	0.01	0.01	0	0	0
**2008**	0.02	0.02	0.94	98.75	0.09	0.02	0.01	0.01	0.01	0.02	0.01	0.01	0.01	0.01	0.01
**2009**	0.02	0.01	0.02	1.04	98.85	0.06	0.02	0.02	0.02	0.01	0.01	0.01	0.01	0.01	0.01
**2010**	0.03	0.02	0.01	0.02	0.99	98.79	0.02	0.02	0.02	0.01	0.01	0.01	0	0.01	0.01
**2011**	0.03	0	0.02	0.01	0.01	1.07	99.10	0.03	0.03	0.03	0.03	0.01	0.02	0.01	0.02
**2012**	0.03	0	0	0.03	0.01	0.01	0.80	99.14	0.04	0.02	0.02	0.02	0.03	0.02	0.01
**2013**	0.03	0	0	0	0.01	0.01	0.01	0.74	98.94	0.04	0.03	0.02	0.02	0.02	0.02
**2014**	0.03	0	0	0	0	0.01	0.01	0.01	0.90	99.17	0.05	0.03	0.03	0.02	0.02
**2015**	0.03	0	0	0	0	0	0	0.01	0	0.69	99.0	0.03	0.03	0.02	0.02
**2016**	0.04	0	0	0	0	0	0	0.01	0.01	0	0.81	99.05	0.05	0.03	0.03
**2017**	0.04	0	0	0	0	0	0	0	0.01	0	0.01	0.78	98.98	0.04	0.04
**2018**	0.04	0	0	0	0	0	0	0	0	0.01	0.01	0	0.78	99.16	0.04
**2019**	0.04	0	0	0	0	0	0	0	0	0	0.01	0.01	0	0.62	99.29

PA=pension authority

Table S5 distribution of baseline (2006) characteristics of the study population according to level of job control

Job control		Men			Women		
		**Low** N=514,114	**Medium** N=510,831	**High** N=522,348	**Low** N=564,977	**Medium** N=479,815	**High** N=519,739
		%	%	%	%	%	%
**Age**	30-39	35.3	32.3	34.5	32.6	30.6	34.9
	40-49	33.7	31.8	32.0	33.3	32.0	32.5
	50-60	31.0	35.9	33.6	34.1	37.4	32.6
**Birth country**	Outside Sweden	19.0	9.6	7.1	18.7	10.0	9.5
**SEP**	Unskilled manual	32.4	27.2	20.0	31.2	25.7	21.5
	Skilled manual	21.1	23.4	17.9	22.5	21.3	18.3
	Lower non-manual	7.4	10.0	13.3	7.4	11.2	12.1
	Intermediate non-manual	10.8	15.8	26.1	10.4	18.7	22.9
	Professional non-manual	2.5	5.1	10.3	2.3	5.6	9.6
	Farmer	5.1	7.1	4.0	5.7	6.1	5.0
	Not classified	20.7	11.3	8.3	20.4	11.3	10.7
**CCI**	Previous diagnosis	6.3	6.0	5.0	6.0	5.9	5.4
**Education**	≤9 years	26.1	16.0	5.9	19.7	5.9	5.0
	10-11 years	45.7	39.3	18.0	49.3	23.9	21.2
	12 years	15.3	16.0	15.8	19.0	13.1	15.5
	13-14 years	7.7	13.0	23.2	7.8	24.3	19.1
	≥15	5.2	15.8	37.1	4.3	32.9	39.2
**Marital status**	Married	43.3	49.9	59.0	50.0	57.6	54.6
	Unmarried	42.5	37.6	30.8	30.6	27.0	29.6
	Divorced	13.7	12.0	9.7	17.4	14.0	14.6
	Widowed	0.5	0.6	0.5	1.9	1.5	1.3
**Children**	0	57.0	53.0	47.5	47.9	45.5	46.0
	1	15.0	16.2	17.2	19.1	18.9	19.7
	2	19.2	22.2	26.4	22.7	25.9	26.3
	3	6.7	7.0	7.7	8.0	8.1	7.0
	4 or more	2.1	1.6	1.2	2.3	1.6	1.1
**Sector**	Private	81.3	70.7	77.2	46.2	28.3	51.7
**PWL**	Low	1.3	16.8	76.7	0	23.6	80.0
	Medium	31.0	50.4	23.3	16.2	70.8	18.6
	High	67.7	32.8	0	83.8	5.6	1.4

SEP=socioeconomic position in childhood, CCI=Charlson co-morbidity index, PWL=physical workload

Table S6 expected average years spent in different states up to 65 years of age and 95% confidence intervals by sex, age, and level of job control without adjustment for covariates

**Men**	**Job control**	**Work**	**Sickness absence**	**Unemployed**	**Other**	**Disability pension**	**Early pension**	**Death**
30	Low	23.73 (23.30-24.16)	1.35 (1.22-1.47)	3.82 (3.70-3.95)	3.58 (3.29-3.88)	1.18 (1.07-1.28)	0.55 (0.52-0.59)	0.60 (0.52-0.68)
	Medium	24.70 (24.27-25.13)	1.47 (1.35-1.60)	2.83 (2.70-2.95)	3.23 (2.94-3.53)	1.34 (1.23-1.44)	0.92 (0.89-0.96)	0.48 (0.40-0.56)
	High	27.76 (27.33-28.19)	0.76 (0.63-0.89)	1.57 (1.44-1.69)	3.37 (3.07-3.66)	0.30 (0.20-0.41)	0.68 (0.64-0.71)	0.46 (0.38-0.54)
40	Low	17.62 (17.19-18.05)	1.01 (0.88-1.13)	2.07 (1.94-2.19)	2.53 (2.23-2.82)	0.90 (0.79-1.01)	0.58 (0.54-0.61)	0.46 (0.38-0.54)
	Medium	18.46 (18.03-18.90)	0.83 (0.71-0.96)	1.51 (1.38-1.63)	2.10 (1.80-2.39)	0.89 (0.78-1.00)	0.92 (0.89-0.95)	0.36 (0.28-0.44)
	High	20.08 (19.64-20.51)	0.50 (0.37-0.63)	0.86 (0.73-0.98)	2.37 (2.07-2.66)	0.18 (0.07-0.29)	0.65 (0.62-0.69)	0.25 (0.17-0.33)
50	Low	10.32 (9.89-10.75)	0.67 (0.55-0.80)	1.08 (0.95-1.20)	1.60 (1.31-1.90)	0.44 (0.33-0.54)	0.55 (0.51-0.58)	0.25 (0.17-0.33)
	Medium	10.59 (10.16-11.02)	0.55 (0.42-0.67)	0.83 (0.71-0.95)	1.20 (0.91-1.50)	0.40 (0.29-0.50)	0.99 (0.95-1.02)	0.15 (0.07-0.23)
	High	11.56 (11.13-11.99)	0.29 (0.17-0.42)	0.39 (0.26-0.51)	1.45 (1.16-1.75)	0.08 (-0.03-0.19)	0.66 (0.63-0.7)	0.13 (0.05-0.21)
60	Low	2.90 (2.47-3.33)	0.18 (0.06-0.31)	0.36 (0.23-0.48)	0.76 (0.47-1.06)	0.08 (-0.03-0.19)	0.62 (0.59-0.66)	0.05 (-0.03-0.13)
	Medium	2.82 (2.39-3.25)	0.17 (0.04-0.29)	0.26 (0.14-0.38)	0.50 (0.21-0.80)	0.08 (-0.03-0.19)	0.98 (0.94-1.01)	0.03 (-0.05-0.11)
	High	3.17 (2.74-3.60)	0.11 (-0.02-0.23)	0.18 (0.05-0.3)	0.69 (0.4-0.99)	0.01 (-0.1-0.12)	0.66 (0.63-0.70)	0.03 (-0.05-0.11)
**Women**								
30	Low	21.51 (21.07-21.94)	2.10 (1.92-2.29)	3.52 (3.22-3.83)	3.70 (3.45-3.95)	3.01 (2.52-3.49)	0.57 (0.53-0.61)	0.31 (0.26-0.36)
	Medium	23.56 (23.12-24.00)	2.06 (1.88-2.25)	2.04 (1.73-2.35)	3.20 (2.95-3.45)	2.82 (2.33-3.31)	0.85 (0.82-0.89)	0.19 (0.13-0.24)
	High	25.23 (24.79-25.67)	1.99 (1.8-2.17)	1.63 (1.32-1.94)	3.38 (3.14-3.63)	1.13 (0.64-1.62)	0.57 (0.53-0.61)	0.22 (0.17-0.27)
40	Low	16.56 (16.12-16.99)	1.54 (1.35-1.72)	1.85 (1.54-2.16)	1.93 (1.69-2.18)	1.04 (0.56-1.53)	0.58 (0.55-0.62)	0.25 (0.20-0.30)
	Medium	18.12 (17.69-18.56)	1.47 (1.29-1.66)	1.03 (0.72-1.34)	1.58 (1.33-1.83)	1.88 (1.39-2.36)	0.91 (0.87-0.94)	0.20 (0.15-0.25)
	High	19.50 (19.06-19.93)	1.21 (1.02-1.40)	0.96 (0.65-1.26)	1.83 (1.58-2.07)	0.66 (0.17-1.15)	0.60 (0.56-0.64)	0.14 (0.09-0.20)
50	Low	10.57 (10.13-11.00)	0.87 (0.68-1.06)	0.88 (0.57-1.19)	1.18 (0.93-1.43)	0.59 (0.1-1.08)	0.66 (0.62-0.70)	0.05 (0.00-0.1)
	Medium	11.04 (10.60-11.47)	0.75 (0.56-0.93)	0.45 (0.14-0.76)	0.90 (0.65-1.15)	0.61 (0.12-1.10)	0.95 (0.91-0.99)	0.07 (0.02-0.12)
	High	11.90 (11.46-12.33)	0.65 (0.47-0.84)	0.44 (0.14-0.75)	1.13 (0.89-1.38)	0.21 (-0.27-0.70)	0.61 (0.57-0.65)	0.07 (0.02-0.12)
60	Low	2.93 (2.50-3.37)	0.24 (0.06-0.43)	0.28 (-0.03-0.59)	0.71 (0.46-0.96)	0.07 (-0.42-0.56)	0.70 (0.66-0.74)	0.01 (-0.04-0.06)
	Medium	2.94 (2.50-3.38)	0.21 (0.03-0.40)	0.15 (-0.15-0.46)	0.49 (0.24-0.74)	0.09 (-0.39-0.58)	0.99 (0.95-1.03)	0.01 (-0.04-0.07)
	High	3.21 (2.78-3.65)	0.17 (-0.01-0.36)	0.16 (-0.15-0.46)	0.66 (0.41-0.90)	0.03 (-0.45-0.52)	0.62 (0.58-0.65)	0.02 (-0.04-0.07)

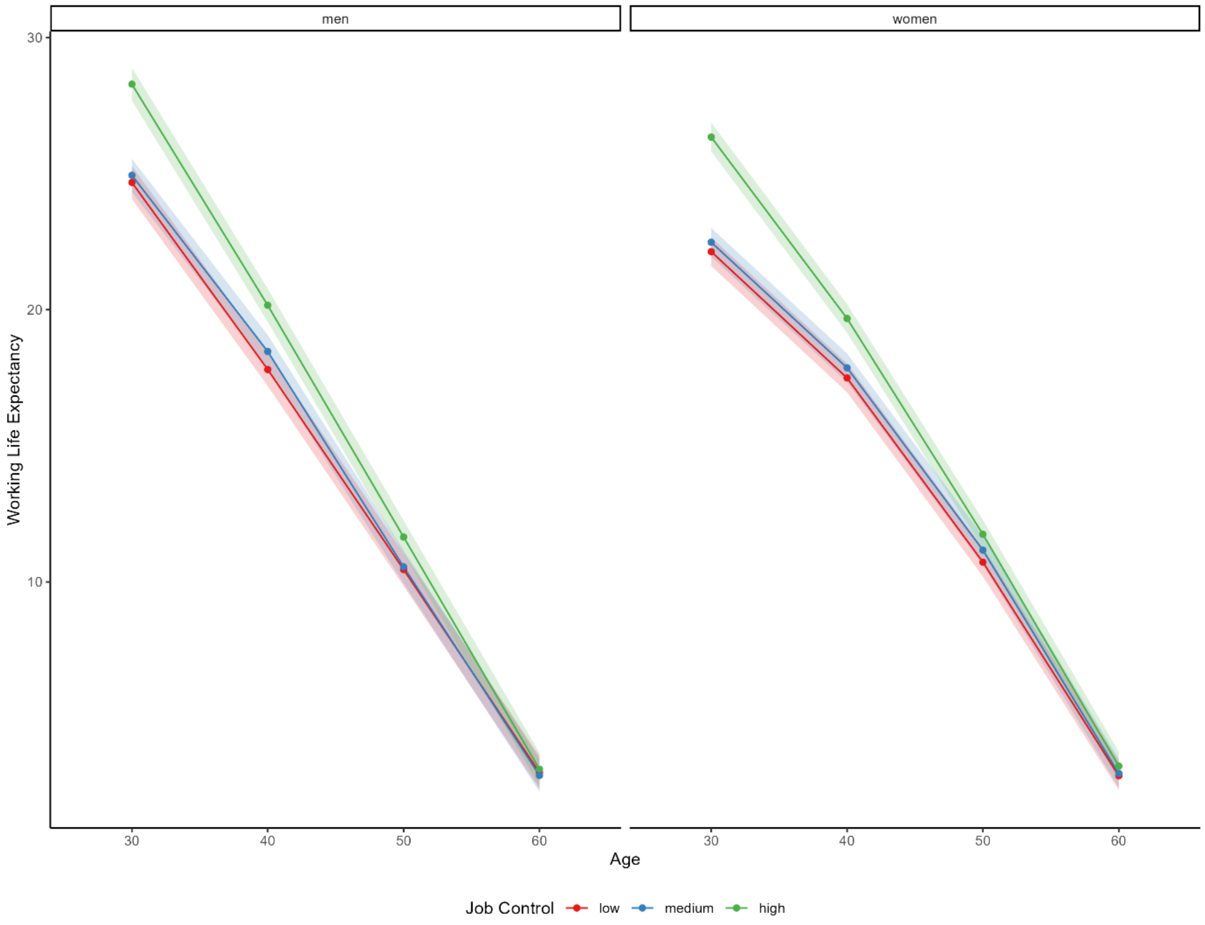

Figure S1 shows working life expectancy and 95% confidence intervals according to sex, age, and level of job control excluding those with high physical workload

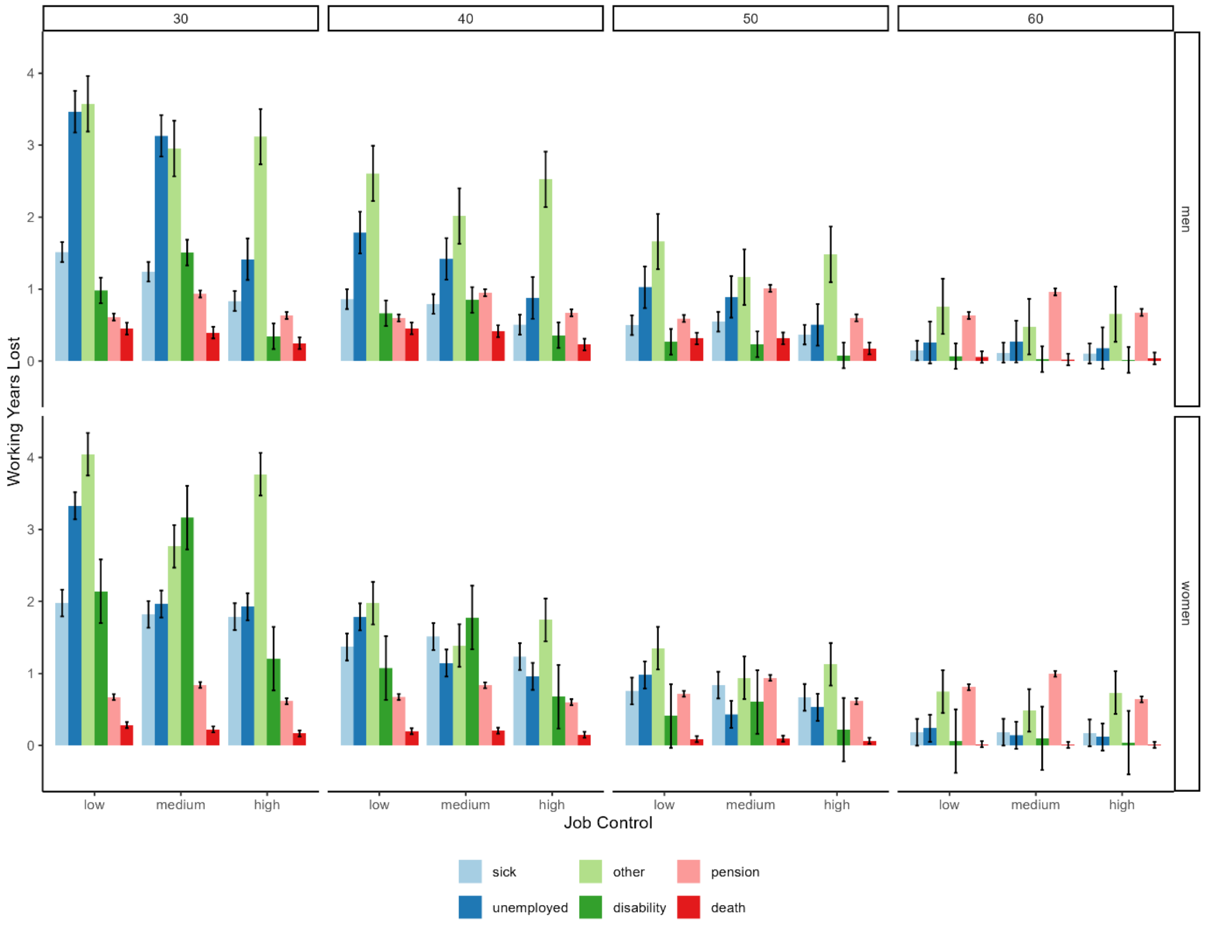

Figure S2 shows the expected working years lost due to sickness absence, unemployment other, disability pension, early old age pension, and death and 95% confidence intervals according to sex, age, and level of job control excluding those with high physical workload
